# Giant Aortic Root Aneurysm Treated With Bentall Procedure: Technical Pearls and Intraoperative Strategies

**DOI:** 10.7759/cureus.88717

**Published:** 2025-07-25

**Authors:** Vasileios Leivaditis, Athanasios Papatriantafyllou, Theodoros Milas, Nikolaos G Baikoussis

**Affiliations:** 1 Department of Cardiothoracic and Vascular Surgery, Westpfalz-Klinikum, Kaiserslautern, DEU; 2 Department of Cardiac Surgery, Ippokrateio General Hospital of Athens, Athens, GRC

**Keywords:** aortic rupture, aortic valve regurgitation, ascending aortic aneurysm, bentall procedure, emergency aortic surgery, giant aortic root, valve replacement

## Abstract

Giant aortic root aneurysms are rare and potentially life-threatening, especially when the diameter exceeds 10 cm. These cases require urgent surgical intervention and pose significant technical challenges due to the risk of rupture, distorted anatomy, and associated valve dysfunction. We report the case of a 58-year-old man who presented with acute chest pain and refractory hypertension. Transthoracic echocardiography revealed severe aortic regurgitation, a dilated left ventricle with an ejection fraction below 30%, and an aortic root estimated at over 11 cm. CT angiography confirmed a giant aortic root aneurysm without dissection. Given the imminent risk of rupture, the patient underwent emergent open surgical repair. Axillary artery cannulation and early heparinization were performed to minimize intraoperative risk. Heparin was administered after axillary artery cannulation and before sternotomy to enable immediate initiation of cardiopulmonary bypass in case of rupture. A standard Bentall procedure using a 27 mm mechanical valved conduit and 30 mm Valsalva graft was successfully completed. The postoperative course was uneventful, and follow-up at three and nine months showed excellent clinical recovery and graft function. This case underlines the importance of early recognition and timely surgical intervention in giant aortic root aneurysms. Although the Bentall procedure is routinely performed worldwide, the surgical management of giant aneurysms >10 cm remains technically complex and underreported. Preoperative planning, alternative cannulation strategies, and meticulous surgical technique are crucial for safe and effective management of these high-risk cases.

## Introduction

Thoracic aortic aneurysm (TAA), most commonly involving the ascending aorta and root, is typically a silent condition with potentially life-threatening complications such as dissection and rupture. TAAs are common, are detected in 10% of autopsies, have an incidence of 5.9 per 100,000 person-years, and are the most common reason for thoracic aortic surgery. The median age at the time of diagnosis is 65 years, and this lesion occurs two to four times more frequently in males [1,2].

The etiology of TAA is multifactorial. Most cases are degenerative in origin, with atherosclerosis and hypertension being major contributing factors [3,4]. Other causes include inflammatory conditions collectively referred to as aortitis. Additionally, a subset of TAAs is syndromic, associated with connective tissue disorders such as Marfan and Ehlers-Danlos syndromes [5].

In this report, we present a rare and dramatic case of a giant aortic root aneurysm, measuring 11 cm in maximal diameter, which was successfully treated with open surgical repair. The case highlights both the complexity of surgical decision-making in such high-risk scenarios and the technical nuances essential for optimal outcomes. While the Bentall procedure is a well-established and globally practiced technique, cases involving aneurysms larger than 10 cm remain extremely rare and carry unique surgical risks. Therefore, the detailed presentation of such cases adds valuable insight to existing literature.

## Case presentation

**Figure 1:**
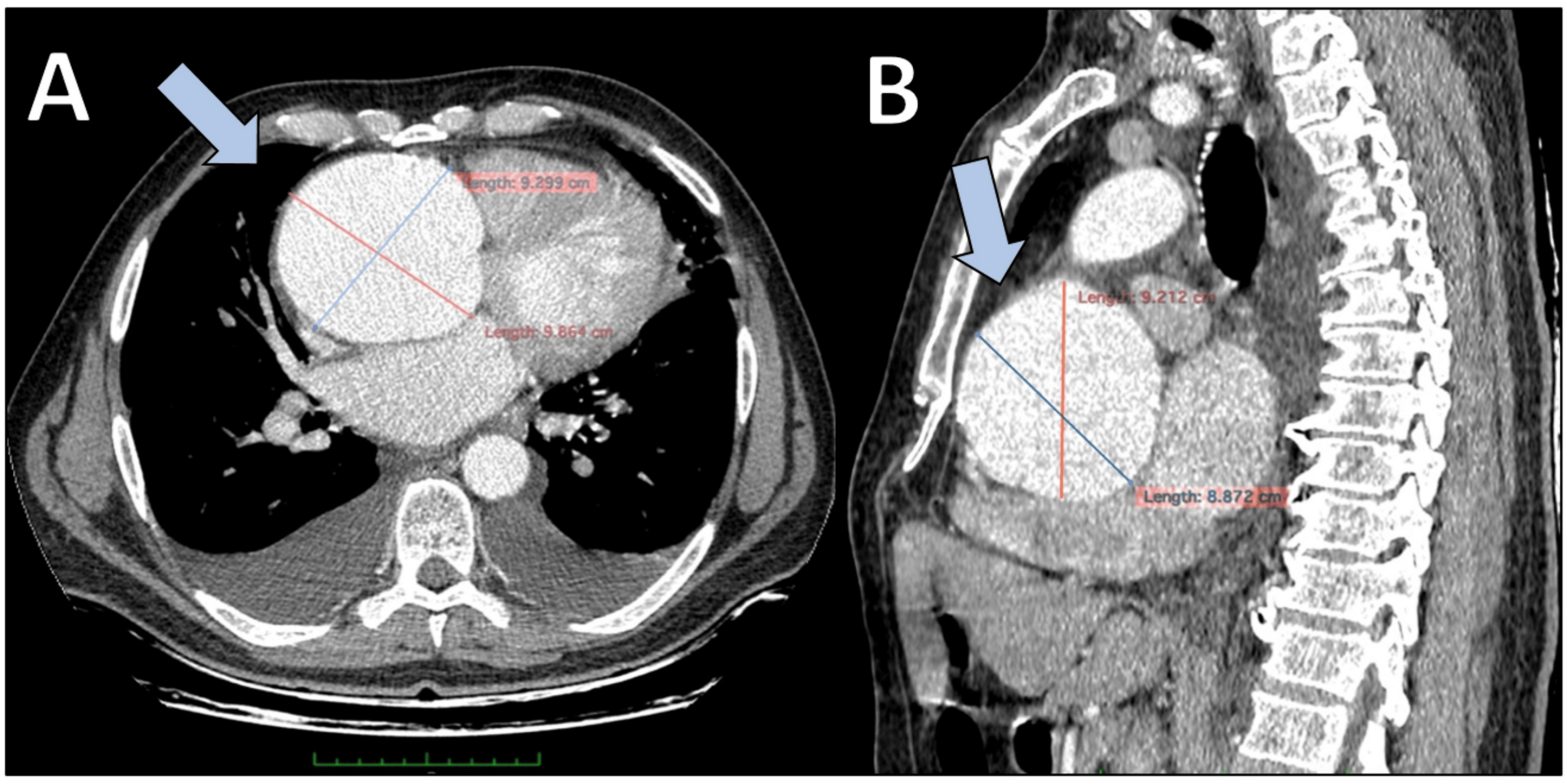
Preoperative CT images of the giant aortic root aneurysm. (A) Axial cross-sectional view demonstrating a massive aneurysmal dilation of the aortic root, measuring over 10 cm in diameter, compressing adjacent mediastinal structures. (B) Sagittal reconstruction highlighting the full extent of the aortic root dilation, with preserved continuity of the ascending aorta and absence of dissection or rupture.

We report the case of a 58-year-old male patient who presented to the emergency department with acute chest pain and persistent hypertension (220/110 mmHg). On physical examination, the patient had a BMI of 27.4 kg/m². He had no skeletal features suggestive of a connective tissue disorder, and he had a 20-pack-year smoking history. Transthoracic echocardiography revealed severe aortic valve regurgitation, a markedly dilated left ventricle with an end-diastolic diameter of 7.0 cm, a reduced left ventricular ejection fraction of less than 30%, and a massively enlarged aortic root estimated to exceed 11 cm in diameter. Due to these alarming findings, a CT of the chest with intravenous contrast was promptly performed. Imaging confirmed the presence of a giant aortic root aneurysm and a significantly dilated left ventricle, with no evidence of acute or chronic aortic dissection (Figure 1).

The patient’s blood pressure remained severely elevated at 220 mmHg despite intravenous administration of nitroglycerin, calcium channel blockers, and esmolol. The chest pain was attributed to mechanical compression of surrounding mediastinal structures by the aneurysmal root and the imminent risk of rupture. Given the clinical instability and imaging findings, the case was deemed emergent, and immediate surgical intervention was undertaken.

Following the induction of general anesthesia, right axillary artery cannulation was performed. A 10 mm synthetic graft was anastomosed to the right subclavian artery to facilitate arterial access for cardiopulmonary bypass. Systemic heparin was administered prior to sternotomy as part of our preventive strategy to ensure that cardiopulmonary bypass could be rapidly established in the event of aneurysmal rupture during chest entry. This approach aimed to minimize catastrophic bleeding and allow immediate circulatory support if needed. Sternotomy was performed using a standard oscillating saw with great care to avoid compression or shear forces over the anterior aneurysmal wall. In this case, alternative sternal entry techniques such as “open pericardium-first” were not required.

A median sternotomy was performed, revealing extensive fibrosis and adhesions surrounding the heart and ascending aorta. Careful and meticulous lysis of adhesions enabled exposure of the right atrium for venous cannulation, followed by dissection of the entire aortic root. Retrograde cardioplegia was not feasible due to dense adhesions, and antegrade cardioplegia through the aortic root vent was also not possible due to severe aortic insufficiency. Therefore, following clamping of the ascending aorta, an aortotomy was performed, and cold blood cardioplegia was directly infused into the coronary ostia to achieve myocardial protection.

**Figure 2:**
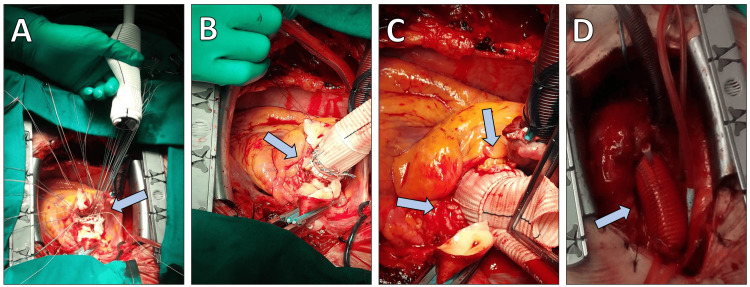
Intraoperative photographs of the surgical procedure. (A) Suturing of the mechanical valved conduit (Carboseal Valsalva graft) to the native aortic annulus. (B) Precise placement of the prosthetic conduit within the aortic root following resection of the aneurysm. (C) Reimplantation of the mobilized coronary artery buttons into the conduit using end-to-side anastomoses. (D) Final view demonstrating the completed Bentall procedure with successful graft placement and coronary reimplantation.

The aneurysmal aortic root was excised, and the coronary ostia were mobilized and prepared as buttons for reimplantation. After appropriate sizing, a composite valved graft was selected: a 27 mm mechanical valve integrated into a 30 mm Valsalva-type conduit (Carboseal Valsalva graft). A standard Bentall procedure was performed, with reimplantation of both coronary buttons into the graft (Figure 2).

The patient’s early postoperative course was uneventful, and he was discharged on the fifth postoperative day. At the three-month follow-up, the patient reported significant improvement in functional status. Imaging studies demonstrated restoration of left ventricular function and appropriate prosthetic valve function. At nine months, he remained fully active, and routine laboratory tests and echocardiography showed no abnormalities or complications.

## Discussion

Open surgical repair remains the gold standard for the management of TAAs, particularly those involving the ascending aorta and root, due to its proven long-term durability and lifesaving potential in acute cases [5-7]. When the aortic root is involved, surgical intervention requires reimplantation of the coronary arteries and either repair or replacement of the aortic valve. In cases where both the ascending aorta and the aortic valve are diseased or anatomically compromised, the modified Bentall procedure, with composite graft replacement of the root and valve, is most commonly employed [6].

Surgical repair of a giant aortic root aneurysm exceeding 10 cm presents unique intraoperative challenges. The massive dilation increases the risk of spontaneous rupture during sternotomy or dissection, compromises myocardial protection strategies, and complicates anatomical orientation. In such scenarios, pre-sternotomy axillary artery cannulation is a crucial safety measure, allowing for rapid initiation of cardiopulmonary bypass in the event of root rupture upon chest entry [7]. In our case, heparin was administered only after axillary cannulation was secured, minimizing the risk of uncontrolled bleeding while ensuring preparedness for emergent bypass. Alternatively, preoperative femoral venous cannulation and early systemic heparinization provide further protection by allowing for prompt circulatory support and hypothermic cooling if needed. Sternal entry was done cautiously using the standard technique, but in more unstable or anteriorly compressed cases, alternative access techniques may be considered. The extent of aneurysmal dilation is a determinant in selecting cerebral protection methods. Our patient’s aneurysm was confined to the root and did not involve the arch, so deep hypothermic circulatory arrest was not required.

One significant technical difficulty in large root aneurysms is the increased distance between the coronary ostia, making reimplantation during a Bentall procedure more demanding. Extensive mobilization of both coronaries is often necessary, and in some cases, interposition grafts (e.g., 8 mm Dacron grafts) may be used to bridge the gap and prevent kinking or tension at the anastomoses [3,6].

Another major consideration in root aneurysm repair is the status of the aortic valve and annulus. In select patients, valve-sparing root replacement is a viable and increasingly favored approach, offering excellent long-term results without the need for lifelong anticoagulation [8]. However, this technique is best suited for patients with preserved cusp anatomy and only mild to moderate annular dilation. When the annulus is severely dilated, valve repair becomes technically challenging, and its durability is questionable. In such cases, annular stabilization techniques (e.g., subannular ring implantation) have been developed, though they may not be applicable in emergency settings with poor ventricular function or gross regurgitation [9].

In our case, the presence of severe aortic regurgitation, markedly depressed left ventricular function, and the urgent nature of the presentation precluded valve-sparing options. A mechanical composite graft was chosen to ensure long-term valve competence and structural integrity of the aortic root.

Finally, intraoperative transesophageal echocardiography plays a critical role in evaluating the adequacy of valve repair or replacement, confirming coronary perfusion, and guiding de-airing maneuvers. Its routine use is strongly recommended in complex root surgery to ensure procedural success and prevent postoperative complications.

In summary, surgical management of giant aortic root aneurysms demands careful preoperative planning, technical precision, and intraoperative flexibility. Familiarity with adjunct techniques, such as alternative cannulation strategies, coronary button mobilization, and graft interposition, is essential for successful outcomes in these high-risk patients.

## Conclusions

Giant aortic root aneurysms exceeding 10 cm are extremely rare and pose significant surgical challenges due to the heightened risk of rupture, anatomical distortion, and technical complexity of repair. Prompt recognition, meticulous surgical planning, and the use of tailored intraoperative strategies - such as alternative cannulation sites and coronary button mobilization - are essential for successful outcomes. The Bentall procedure remains a reliable and definitive solution in cases of root aneurysm with significant aortic valve insufficiency. This case highlights the feasibility and safety of open surgical repair, even in extreme anatomical scenarios, when approached with appropriate expertise and technique. Although the Bentall operation is widely practiced, careful adaptation is required in giant aneurysms, and sharing such rare cases contributes to collective surgical experience.
